# Direct-to-consumer genetic testing: advantages and pitfalls

**DOI:** 10.5808/GI.2019.17.3.e33

**Published:** 2019-09-26

**Authors:** Bermseok Oh

**Affiliations:** Department of Biochemistry and Molecular Biology, Kyung Hee University School of Medicine, Seoul, Korea

## What is Direct-to-Consumer Genetic Testing?

Conventional genetic testing is a process in which clinicians take samples from patients as clinically needed, send them to a laboratory for genetic testing, and discuss the test results with patients. Direct-to-consumer (DTC) genetic testing is different from conventional genetic testing in that consumers learn about DTC genetic testing from TV commercials, the internet, or in-store advertising, and request the tests to be performed by DTC companies by their own choice. If conventional genetic testing is a clinician-centric framework, then DTC genetic testing is a consumer-centric one.

Normally, saliva or mouth cells obtained by scratching the inside of one’s mouth with a cotton swab are sent to the DTC company. The company isolates DNA from the sample and conducts a genetic test. The test results are analyzed through proprietary in-house programs and a genetic profile report of various phenotypes is sent to the consumer. The test items or test report may vary depending on the needs of the consumer, as well as operational characteristics of the company.

Conventional clinician-centric tests are aimed at diagnosing and treating patients’ diseases, mainly limited to genetic diseases or cancers. Consumer-centric DTC tests do also carry out genetic tests for diseases, but have an additional emphasis on biometric/life-related concerns, such as obesity, nutrition, skin, hair loss, ancestry, and life cycle. Therefore, the aim of DTC genetic testing is to determine consumers’ propensity for such phenotypes or genetic predisposition to the tested diseases for preventive purposes, rather than for the diagnosis and treatment of diseases. The cost of DTC genetic testing varies from company to company, but is roughly in the range of 100–1,000 US dollars, and DTC genetic testing is generally not covered by insurance.

## What Are the Advantages and Disadvantages of DTC Genetic Testing?

The advantages of DTC genetic testing can be described as follows [1,2]. First, DTC genetic testing may provide an opportunity for consumers to recognize the importance of genetics in diverse phenotypes, including diseases. If the test results indicate that a consumer has a genetic predisposition to a certain disease or phenotype of interest, then he or she may take proactive steps to improve his or her own health. It is easy to access genetic information through DTC testing because it does not require approval from clinicians or insurance companies. Moreover, DTC genetic testing is generally less expensive and faster than genetic testing performed at hospitals. The sample is normally collected non-invasively, such as through gathering saliva or scratching the inside of one’s mouth with a cotton swab, instead of drawing blood, as is performed at the hospital. Lastly, the data from consumers’ genetic tests can be collected into valuable databases to help with research in related fields, as shown by the papers published by 23andMe, one of the leading DTC genetic testing companies in the United States [3,4].

Nonetheless, DTC genetic testing has important drawbacks [1,2]. DTC genetic testing often does not provide conclusive results on whether the consumer will develop a disease or not. Most genetic tests performed by DTC companies are limited to few major genetic variants related to the phenotypes of interest, which leads to poor discriminatory power. Diseases are generally affected by many genetic variants—in other words, they are polygenic. In addition to genetic factors, disease incidence is influenced by environmental and lifestyle factors including age, sex, race, nutrition, exercise, and stress. Thus, DTC genetic testing itself does not guarantee that a consumer with a high genetic risk score will suffer from a certain disease. Instead, it only indicates that one has a genetic propensity for that disease. If one is affected by favorable environmental factors and has a lifestyle that is beneficial for that disease, one may not develop the disease despite a high genetic risk. On the contrary, a consumer with a low genetic risk may get sick if he or she lives in a disease-prone environment or has lifestyle factors that increase susceptibility to the disease. For example, 23andMe conducts an *APOE* genetic test in relation to Alzheimer disease. The average likelihood of developing Alzheimer disease in carriers of the relevant allele is more than twice as high than in people who do not carry it. However, not everyone with the *APOE* e4 gene will develop Alzheimer disease, and having the *APOE* e2 gene, which confers resistance to Alzheimer disease, is likewise not a guarantee that one will never get Alzheimer disease. Consumers sometimes experience stress when they receive unexpected test results, especially if the results are related to serious diseases, such as cancer [5]. It is recommended that the results of cancer genetic testing be examined after consultation with clinicians, since the genetic test results related to cancer may have significant impacts on consumers. There are also many different genetic variants that are related to a specific cancer but not available for DTC genetic testing. For example, only three genetic variants are examined for genetic testing of *BRCA1* and *BRCA2* conducted by 23andMe with U.S. Food and Drug Administration approval. However, as many as 1,000 mutations of *BRCA1* and *BRCA2* are known to affect the risk of breast and ovarian cancer. Furthermore, family history is known to explain only about 5%–10% of cancer cases. A wide variety of factors, including age, sex, nutrition, exercise, race, disease history, hormonal factors, and reproductive factors, can affect the development of cancer. Consumers may make decisions on their own with inaccurate or non-deterministic DTC results, and take actions that can damage their health without appropriate consultation with clinicians. Lastly, there is often a lack of scientific evidence for the genetic tests carried out by DTC companies, and consumers’ genetic information may be used for other purposes without their approval or might even be stolen due to inappropriate security measures.

## How Do We Compensate for the Shortcomings of DTC Genetic Testing?

The discovery of genetic variations in diseases or phenotypes of interest (e.g., weight, hair loss, intelligence, etc.) is quickly paying off. Clinical applications have focused on predictive models of disease. Previously, disease predictions were made using a few genetic variants that showed significance in genome-wide association studies (GWASs), on which basis a polygenic score (PGS) was calculated. Most predictions resulted in poor discrimination and imprecision. Predictive models are gradually starting to use more genetic variants, as GWASs are discovering more genetic variations as a consequence of using an increased number of samples, and recently developed predictive models take into account genome-wide variations, calculating a genome-wide polygenic score (GPS). In a study using a GPS for body mass index, the people with the highest score category had an obesity prevalence rate four times higher than that of the other categories [6]. Thus, the GPS method has yielded better predictions than ever before.

The most worrisome factor of all relates to the reporting of genetic test results. The results of DTC genotyping should be clearly communicated to the consumer; in particular, clear guidance should be given regarding what these tests indicate about their illness or health and what they cannot show, with special emphasis on the fact that these tests cannot be a diagnosis of disease. Consumers may make decisions on their own based on inaccurate or non-deterministic DTC results, and may even take actions that can damage their health without appropriate consultation with clinicians. DTC companies should therefore inform consumers of the limitations of the test results and encourage consumers to consult with clinicians before taking action.

Many of the concerns mentioned above as shortcomings of DTC genetic testing remain pending. At this point, the guidelines provided by the American College of Medical Genetics and Genomics for DTC genotyping are as follows [7]. First, genetic testing and the interpretation of results are complex processes. Thus, genetic testing should be performed in a laboratory that has been inspected by an appropriate agency, such as the CLIA program in the United States. In addition, genetic experts, such as clinical geneticists or genetic counselors, should handle requests from consumers and the provision of test results. They will protect consumers from improper informed consent procedures, a lack of pre-test descriptions, incorrect genetic test items, and inappropriate precautions or medical practices resulting from the misinterpretation of test results. Second, consumers should be informed by the DTC company of what the test results can and cannot do before DTC genotyping. Third, the DTC company should explain that unexpected, or unrequested, results may come from genotyping. Consumers should also be informed that these unforeseen consequences can affect not only themselves, but also their family members. Fourth, the DTC company should inform the consumer about the scientific basis upon which the genetic test was conducted. If this is too technical for the general public to understand, it should be explained in a way that facilitates easy understanding. Fifth, consumers should receive the following information as part of an explanation about personal information protection: who will see the results of a consumer’s genetic test, what measures will be taken to protect the genetic information, how the sample will be processed after it is used, how the genetic test results will subsequently affect life insurance or disability insurance, who owns the genetic information produced, and whether the genetic information can be provided to third parties.

## Conclusion

DTC genetic testing provides consumers with the opportunity to learn about their genetic profiles related to phenotypes of interest in a convenient and less expensive manner. Furthermore, the list of test items will increase and the predictive accuracy will be improved as related research continues to progress rapidly. However, many of the concerns previously mentioned as shortcomings of DTC genetic testing remain pending. While DTC companies should try to compensate for these shortcomings themselves to the extent possible, regulation over DTC companies should be established by the appropriate agency to safeguard consumers from the abuse of DTC genetic testing.
